# csDMA: an improved bioinformatics tool for identifying DNA 6 mA modifications via Chou’s 5-step rule

**DOI:** 10.1038/s41598-019-49430-4

**Published:** 2019-09-11

**Authors:** Ze Liu, Wei Dong, Wei Jiang, Zili He

**Affiliations:** 1 College of Water Resources and Architectural Engineering, Northwest A&F University, Yangling, 712100 Shaanxi China; 2 Key Laboratory of Agricultural Soil and Water Engineering in Arid and Semiarid Areas, Ministry of Education, Northwest A & F University, Yangling, 712100 Shaanxi China

**Keywords:** Computational models, Machine learning

## Abstract

DNA N^6^-methyldeoxyadenosine (6 mA) modifications were first found more than 60 years ago but were thought to be only widespread in prokaryotes and unicellular eukaryotes. With the development of high-throughput sequencing technology, 6 mA modifications were found in different multicellular eukaryotes by using experimental methods. However, the experimental methods were time-consuming and costly, which makes it is very necessary to develop computational methods instead. In this study, a machine learning-based prediction tool, named csDMA, was developed for predicting 6 mA modifications. Firstly, three feature encoding schemes, Motif, Kmer, and Binary, were used to generate the feature matrix. Secondly, different algorithms were selected into the prediction model and the ExtraTrees model received the best AUC of 0.878 by using 5-fold cross-validation on the training dataset. Besides, the ExtraTrees model also received the best AUC of 0.893 on the independent testing dataset. Finally, we compared our method with state-of-the-art predictors and the results shown that our model achieved better performance than existing tools.

## Introduction

DNA N^6^-methyldeoxyadenosine (6 mA) modifications were first discovered in *Bacteria* in 1955^1^. However, it had not received much attention as 5-methylcytosine (5mC) did. One important reason is that 6 mA modifications were thought to be only widespread in prokaryotes and unicellular eukaryotes, but rarely in multicellular eukaryotes^2,3^. Researchers have proposed several experimental methods to identify 6 mA modifications in the past few decades. The first method, developed by Dunn *et al*. in 1955, is a combination of ultraviolet absorption spectra, electrophoretic mobility, and paper chromatographic movement, but this method is relatively insensitive and cannot be used to detect 6 mA modifications in animals^1^. Then a restriction enzyme method was used to discover 6 mA modifications in 1978. However, this method can only find modified adenosines that occurred in the restriction enzyme target motifs^4^. With the development of high-throughput sequencing technology, thousands of 6 mA modifications were found in different multicellular eukaryotes. In 2015, Fu *et al*. found 6 mA modifications in 84% genes of *Chlamydomonas* by using 6 mA immunoprecipitation sequencing (6mA-IP-Seq)^5^. In 2016, Koziol *et al*. used dot blots, HPLC, and methyl DNA immunoprecipitation followed by sequencing (MeDIP-seq) to detect 6 mA modifications in vertebrates including *Xenopus laevis*, *mouse* and *human*^6^. In 2017, Mondo *et al*. observed that up to 2.8% of all adenines were methylated in early-diverging *fungi* by using single-molecule real-time (SMRT) sequencing^7^. In 2018, Zhou *et al*. found that about 0.2% of adenines in the *rice* genome were 6 mA methylated by using mass spectrometry, immunoprecipitation, and SMRT, and Zhang *et al*. observed that the 6 mA distribution in the *rice* and *Arabidopsis* genome were very similar by using 6mA-IP-seq^8,9^. As the experimental methods are time-consuming and costly, researchers are trying to predict DNA 6 mA modifications by using computational methods. Two prediction tools are reported up to now, i.e., iDNA6mA-PseKNC^10^ and i6mA-Pred^11^. iDNA6mA-PseKNC is the first prediction tool for predicting 6 mA modifications in the *Mus musculus* genome and i6mA-Pred is the first identification method in the *rice* genome.

Predicting DNA 6 mA modifications based on computational algorithms is still in the infancy. However, in the parallel study of prediction of post-translational modification (PTM) sites, there are many PTM-predicting papers published by the previous researchers^12–22^. Although there is some detailed difference for each of the individual PTMs, the basic core is about the same. Thus, the feature extraction and classification methods proposed in these studies provide a valuable basis for the prediction of DNA 6 mA modifications. In this research, we aim to develop a prediction tool that can be used to predict DNA 6 mA modifications across species. The benchmark datasets created in the iDNA6mA-PseKNC and i6mA-Pred predictors were used and different algorithms were implemented to generate the final optimized model. 5-fold cross-validation was performed and the prediction results demonstrated that our model achieved a better performance than existing 6 mA prediction tools.

As demonstrated by a series of recent publications^10,13–19^ and summarized in two comprehensive review papers^23,24^, to develop a really useful predictor for a biological system, one needs to follow Chou’s 5-steps rule (more detailed description can be found in https://en.wikipedia.org/wiki/5-step_rules.) to go through the following five steps: (1) construct a gold standard dataset to train and test the model; (2) encode samples with effective formulations; (3) conduct the prediction model with a powerful classifier; (4) evaluate model performance by using cross-validation tests and standard measures; (5) establish a user-friendly web-server for the predictor that can be accessible to the public. Below, we are to address these points one by one, making them crystal clear in logic development and completely transparent in operation.

## Method

### Dataset generation

Feng *et al*. created a DNA 6 mA benchmark dataset of the *M. musculus* genome in 2018^10^. The benchmark dataset includes 1,934 positive samples and 1,934 negative samples. Chen *et al*. launched a 6 mA benchmark dataset of the *rice* genome in 2019^11^. The benchmark dataset consists of 880 positive samples and 880 negative samples. The above two benchmark datasets were used to create the cross-species dataset and the CD-HIT-EST software^25^ with different threshold was used to reduce sequence redundancy in the original datasets (Table 1). Finally, the cross-species dataset consists of 2,768 positive samples and 2,716 negative samples with the most rigorous threshold at 0.80, and the length of each sample is 41nt. To build a cross-species 6 mA prediction model, the stratified selection method was used and we random selected 80% samples for model training and the left 20% for independent testing. Finally, the training dataset consists of 2,214 positive samples and 2,214 negative samples, while the independent testing dataset includes 554 positive samples and 502 negative samples.

**Table 1:** Reduce sequence redundancy in the different datasets by using the CD-HIT-EST software.

Species	Dataset	Sequence identity threshold			
		0.95	0.90	0.85	0.80
Mouse	Positive	1,931	1,924	1,914	1,892
	Negative	1,885	1,866	1,844	1,836
Rice	Positive	880	879	878	876
	Negative	880	880	880	880
cross-species	Positive	2,811	2,803	2,792	2,768
	Negative	2,767	2,746	2,724	2,716

### Feature encoding scheme

To construct a DNA 6 mA predictor, one of the most important but also most difficult issue is how to encode feature vector for each sequence, yet still retains most of the key patterns. The pseudo amino acid composition (PseAAC) was proposed by Chou *et al*. and has been widely used in nearly all the areas of computational proteomics^26,27^. Based on the PseAAC, four powerful software, such as ‘PseAAC’^28^, ‘PseAAC-Builder’^29^, ‘propy’^30^, and ‘PseAAC-General’^31^, were established: the former three are for generating various modes of Chou’s special PseAAC^32^; while the 4th one for those of Chou’s general PseAAC^23^. Encouraged by the successes of using PseAAC to deal with protein/peptide sequences, the concept of Pseudo K-tuple Nucleotide Composition (PseKNC)^33^ was developed for encoding features of DNA/RNA sequences^34–36^ that have proved very useful as well. Particularly, recently a very powerful web-server called ‘Pse-in-One’^37^ and its updated version ‘Pse-in-One2.0’^38^ have been established that can be used to generate any desired feature vectors for protein/peptide and DNA/RNA sequences according to the need of users’ studies.

### K-mer pattern

*K* monomeric units (*k*-mers), are simply patterns of *k* consecutive nucleic acids^37^ and have a total of 4^*k*^ kinds of nucleotide patterns for DNA/RNA. Such as 1-mer has 4 and 2-mer has 16 kinds of nucleotide patterns. To calculate the frequencies of *k*-mer nucleotide patterns, the length range *L* of the scanning region must be determined at first, and then the absolute frequencies of the *k*-mer nucleotide patterns are calculated from the start position to the *L-k*-1 position. Finally, the relative frequencies of *k*-mer patterns are calculated for each region. In this study, we set *k* as 2, 3, 4, and extracted 4^2^ + 4^3^ + 4^4^ = 336 kinds of *k*-mer nucleotide patterns for feature encoding.

### KSNPF frequency

The KSNPF frequencies are nucleotide pairs separated by *k* arbitrary nucleotides and have been successfully employed for the prediction of mucin-type O-glycosylation sites^39^ and phosphotase-specific dephosphorylation sites^40^. The KSNPF can be calculated using the following equation:

1 $$f(n1Gap(k)n2)=\frac{{\rm{S}}({\rm{n}}1{\rm{Gap}}({\rm{k}}){\rm{n}}2)}{{\rm{L}}-{\rm{k}}-1}$$

where *n*1 and *n*2 represent a pair of sequence elements. For nucleotide, *n* stands for any one of A, C, G, T/U. Thus, there are 4^2^ = 16 combinations in each pair. *Gap*(*k*) stands for *k* arbitrary elements at intervals and S(n1Gap(k)n2) indicates the number of occurrences of the element pair. In this study, *L* represents the length of the nucleotide sequence, and the *k* was set as 1, 2, 3, 4, and the dimension of the KSNPF can be calculated by 4^2^ × 4 = 64.

### Nucleic shift density

Nucleic shift density encoding can be used to calculate the density of any nucleotide at the current position in its prefix string and has been used to encode nucleotide sequences in the iDNA6mA-PseKNC predictor^10^. A nucleic shift density feature at any position can be defined as follows:

2 $${d}_{i}=\frac{1}{{N}_{i}}{\sum }_{j=1}^{i}F({n}_{j}),\,F({n}_{j})=\{\begin{array}{c}1\,if\,{n}_{j}=q\\ 0\,other\,case\end{array}$$

where *q* represents of any nucleotide at current position *i*, *N*_*i*_ is the length of the ith prefix string in the sequence. For example, the DNA sequence “CAGCTG”. The Nucleic shift density of ‘C’ at the position 1, 2, 3, 4, 5 or 6 is 1/1 = 1, 1/2 = 0.5, 1/3 ≈ 0.33, 2/4 = 0.5, 2/5 = 0.4 or 2/6 ≈ 0.33, respectively. In this study, the length of each sample is 41nt. Thus, 41 Nucleic shift density features were generated for each sample.

### Binary code

Binary encoding scheme is used to predict 6 mA modifications in the iDNA6mA-PseKNC predictor^10^. For the nucleotide in position *i*, the Binary features can be defined as following:

3 $$\{\begin{array}{c}{x}_{i}=\{\begin{array}{c}1\,if\,{n}_{i}\in \{A,G\}\\ 0\,if\,{n}_{i}\in \{C,T\}\end{array}\\ {y}_{i}=\{\begin{array}{c}1\,if\,{n}_{i}\in \{A,T\}\\ 0\,if\,{n}_{i}\in \{C,G\}\end{array}\\ {z}_{i}=\{\begin{array}{c}1\,if\,{n}_{i}\in \{A,C\}\\ 0\,if\,{n}_{i}\in \{G,T\}\end{array}\end{array}$$

In this research, the Binary encoding scheme generates a vector with 3 × 41 = 123 elements by characterizing each nucleotide, “A”, “C”, “G”, or “T”, with (1, 1, 1), (0, 0, 1), (1, 0, 0), or (0, 1, 0), respectively.

### Motif score matrix

The MEME Suite (http://meme-suite.org/) consists of several motif-based sequence analysis tools. In this study, the MEME tool with differential enrichment mode was used and the maximum number of motifs was set to 10. The most enriched motifs were selected based on E-value and the motif matrixes were used for generating motif scores of each sample.

### Performance evaluation

Five different classifiers, Random Forest, GradientBoosting, AdaBoost, ExtraTrees and SVM, were implemented by using Python. For Random Forest, GradientBoosting, AdaBoost, ExtraTrees Classifiers, 1,000 trees were selected for each of them. For SVM, grid research was used to search the best combination of *C* and *gamma* parameters. 5-fold cross-validation was used to evaluate the performance of our model. In a different fold of cross-validation, each subset was iteratively selected as a testing set, while the left 4 subsets were used to train the model. The mean results of the five experiments were finally used as the performance estimates of the algorithms.

Based on the Chou’s symbols introduced for studying signal peptides^41,42^, Four standard measures were derived and have been adopted by several recent publications^43–45^. The measures can be defined as follows:

4 $$\{\begin{array}{c}\begin{array}{l}Sn=1-\frac{{N}_{-}^{+}}{{N}^{+}}\\ Sp=1-\frac{{N}_{+}^{-}}{{N}^{-}}\end{array}\\ ACC=1-(\frac{{N}_{-}^{+}+{N}_{+}^{-}}{{N}^{+}+{N}^{-}})\\ MCC=\frac{1-(\frac{{N}_{-}^{+}}{{N}^{+}}+\frac{{N}_{+}^{-}}{{N}^{-}})}{\sqrt{(1+\frac{{N}_{+}^{-}-{N}_{-}^{+}}{{N}^{+}})(1+\frac{{N}_{-}^{+}-{N}_{+}^{-}}{{N}^{-}})}}\end{array}$$

where *N*^+^ and *N*^−^ refer to the number of positive samples or negative samples, respectively. ${N}_{-}^{+}$ stands for the number of positive samples that were predicted to be negatives, ${N}_{+}^{-}$ refers to the number of negative samples that were predicted to be positives. However, these measures are valid only for single-label learning issues. For the multi-label learning problems, whose appearances are more common in system biology^46^, system medicine^47^ and biomedicine^16^, a completely different set of standard measures is needed^48^. Besides, the receiver operating characteristic curve (ROC) combined with the area under the ROC curve (AUC), the Precision-Recall curve combined with the average precision (AP), and the F1 score^49^ were also used to evaluate the performance of different classifiers.

**Figure 1:**
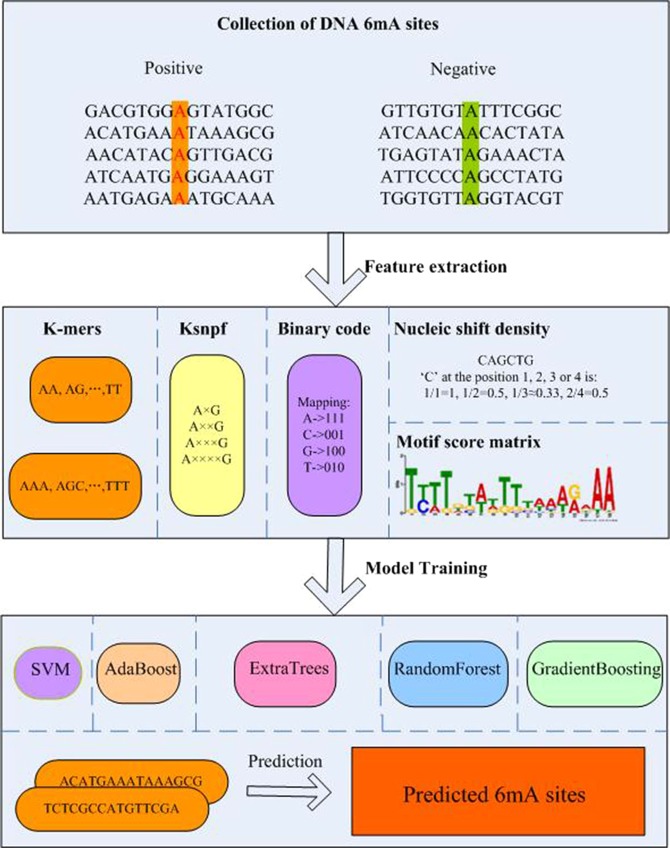
The framework of csDMA.

Using graphic approaches to study biological and medical systems can provide an intuitive vision and useful insights for helping analyze complicated relations therein as shown in the systems of enzyme fast reaction^50^, graphical rules in molecular biology^51^, and low-frequency internal motion in biomacromolecules (such as protein and DNA)^52^. Particularly, what happened is that this kind of insightful implication has also been demonstrated in^53^ and many follow-up publications^54–56^. The framework of csDMA is shown in Fig. 1.

As pointed out by Chou *et al*.^57^ and demonstrated in a series of recent publications^16–18^, publicly accessible web-servers or online bioinformatics tools have significantly increased the impacts of bioinformatics on medical science^58^, driving medicinal chemistry into an unprecedented revolution^59^. Accordingly, the datasets and online tool involved in this paper are all available at https://github.com/liuze-nwafu/csDMA.

## Results

### Differential enrichment motifs discovery

To find the enriched motifs in the flank of 6 mA sites, the MEME tool with differential enrichment mode was used and the maximum number of motifs was set to 10. We used the positive samples in the cross-species dataset as the input and treated the negative samples as the control sequences. The detailed information of the enriched motifs can be found in the supplementary materials. Consider the statistical significance of the motifs, the E-value lower than 0.05 was used to find the most statistically significant motifs and two motifs were selected. The first motif, NNNNNNNHHNHHNHWNTNTNWNNNWNYNNNNNNNNNNNNNN, with an E-value of 3.3e-18 was the most statistically significant. And the third motif ACCGATCSA, with an E-value of 2.9e-2, was also selected. The probability matrixes can also be downloaded from the MEME website which can be used to build motif score matrixes in the training process.

### Model training with different feature subsets

**Figure 2:**
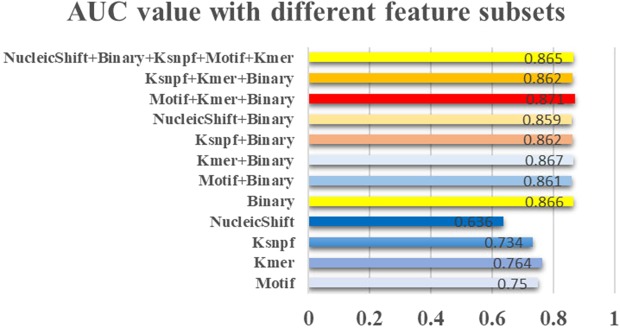
Model performance based on the different feature subsets. 1,000 decision trees were selected into the Random Forest classifier and 5-fold cross-validation was used to evaluate the performance of csDMA.

To find the best combination of feature subsets, different feature subsets were selected into the Random Forest classifier and 5-fold cross-validation was used on the training dataset to evaluate the performance of our model. As shown in Fig. 2, the classifier received an AUC value of 0.866 only by using the Binary code features, which means that the Binary code features were the most significant features that can be used to distinguish positive samples from negative samples. Interestingly, this result was even slightly higher than using combined feature subsets, such as Motif and Binary, Ksnpf and Binary, which achieved an AUC value of 0.861 and 0.862, respectively. Besides, the model achieved the best AUC value of 0.871 when three feature subsets Motif, Kmer, and Binary feature subsets were selected into the classifier. This result was even a little better than the model performance by using all feature subsets. Thus, we used the Motif, Kmer, and Binary encoding scheme to generate the optimized feature matrix.

### Performance evaluation with different classifiers

**Figure 3:**
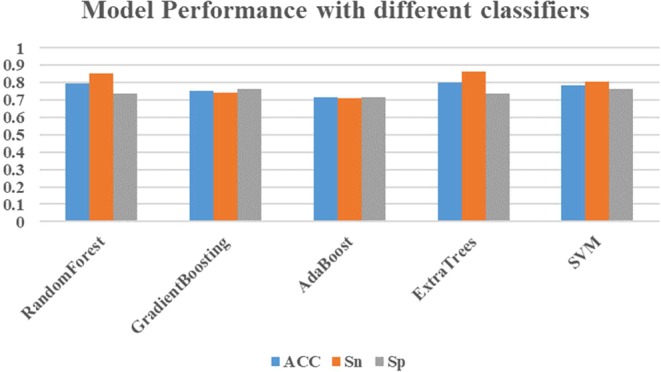
The model performance of different classifiers. The Motif, Kmer, and Binary feature subsets were selected into each classifier and the optimized parameters were used for model training. To evaluate the performance of each classifier, 5-fold cross-validation was used and Standard measures such as ACC, Sn and Sp were used to evaluate the performance of our model.

Five different algorithms were implemented in this research. For the Random Forest, GradientBoosting, AdaBoost, ExtraTrees Classifiers, 1,000 trees were selected for each of them. For the SVM classifier, grid research was used to search the best combination of *C* and *gamma* parameters and the SVM classifier achieved the best performance with *C* of 0.98 and *gamma* of 0.01. To compare the performance of different classifiers, 5-fold cross-validation was used and each classifier was trained with the same fold. As shown in Fig. 3, the ExtraTrees classifier received the best ACC of 0.799 and Sn of 0.864, while the AdaBoost got the lowest ACC of 0.715, Sn of 0.713, Sp of 0.718. However, the ExtraTrees classifier performed not very well for predicting negative samples and received an Sp of 0.735, but it is only a little lower than those of other methods. A more detailed comparison of different classifiers is also shown in Table 2. What’s more, the ExtraTrees classifier also achieved the highest MCC of 0.603, AUC of 0.878 and F1 of 0.811. Thus, we used the ExtraTrees algorithm to train the optimized model.

**Table 2:** Model performance of each algorithm on the training dataset.

Algorithm	Sn	Sp	ACC	MCC	AUC	F1
RandomForest	0.853	0.735	0.794	0.593	0.871	0.806
GradientBoosting	0.743	0.762	0.752	0.506	0.818	0.750
AdaBoost	0.713	0.718	0.715	0.431	0.777	0.715
ExtraTrees	**0.864**	0.735	**0.799**	**0.603**	**0.878**	**0.811**
SVM	0.807	**0.764**	0.785	0.572	0.858	0.790

The highest value of each column is marked in bold.

The independent testing dataset was also used to further evaluate the performance of each classifier. Each classifier was trained on the whole training dataset and evaluated on the independent testing dataset. As shown in Table 3, the ExtraTrees classifier received the best Sn of 0.888, AUC of 0.893 and F1 of 0.832, while the SVM model got the highest Sp of 0.761. Interestingly, the performance of each classifier on the independent testing dataset was even a little higher than that on the training dataset, which suggests that the classifier will receive better performance with a larger training dataset.

**Table 3:** Model performance of the different algorithms on the independent testing dataset.

Algorithm	Sn	Sp	ACC	MCC	AUC	F1
RandomForest	0.875	0.747	**0.814**	**0.630**	0.884	**0.832**
GradientBoosting	0.765	0.757	0.761	0.522	0.854	0.771
AdaBoost	0.776	0.719	0.749	0.496	0.814	0.764
ExtraTrees	**0.888**	0.729	0.813	0.628	**0.893**	**0.832**
SVM	0.843	**0.761**	0.804	0.607	0.875	0.819

The highest value of each column is marked in bold.

### Comparison with existing 6 mA predictors

**Figure 4:**
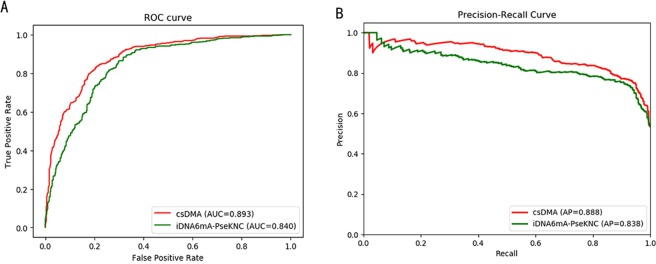
Performance comparison of csDMA and iDNA6mA-PseKNC. (**A**) The ROC curves of csDMA and iDNA6mA-PseKNC. (**B**) The Precision-Recall curves of csDMA and iDNA6mA-PseKNC.

The SVM-based tool iDNA6mA-PseKNC was also implemented in this research. Grid research was used to find the best *C* and *gamma*, and the iDNA6mA-PseKNC achieved the best performance with *C* of 0.336 and *gamma* of 0.02. The same fold used for training csDMA was also used for training iDNA6mA-PseKNC. The iDNA6mA-PseKNC predictor received Sn of 0.767, Sp of 0.769, ACC of 0.767, MCC of 0.536, and F1 of 0.767. Most of the measures were lower except Sp is higher than our model with the ExtraTrees classifier. To further compare the performance of the two algorithms. The ROC and Precision-Recall curves were also plotted in Fig. 4. Our model received an AUC of 0.893, while iDNA6mA-PseKNC got an AUC of 0.840, which also demonstrates that our model achieved better performance than the iDNA6mA-PseKNC predictor.

To test the performance of our model across species, we compared the performance of csDMA and iDNA6mA-PseKNC on the different datasets, i.e., Cross-species, *rice*, and *M. musculus* datasets. For each dataset, 5-fold cross-validation was performed and the previously optimized parameters were used. We used the same fold for training on different datasets. The five-round results of each measure were averaged and shown in Table 4. For the Cross-species dataset, iDNA6mA-PseKNC got an AUC of 0.844, while our model received a higher AUC of 0.879. For the *rice* dataset, iDNA6mA-PseKNC received an AUC of 0.896, while our model achieved a higher AUC of 0.923. For the *M. musculus* dataset, both models got the same AUC values, but our model also received higher MCC and F1 than those of iDNA6mA-PseKNC. All these results show that the proposed method is very accurate and can be used to predict 6 mA sites in different species.

**Table 4:** Model performance of each algorithm across species.

Algorithm	Species	Sn	Sp	ACC	MCC	AUC	F1
csDMA	Cross-species	0.863	0.735	0.799	0.603	0.879	0.811
	Rice	0.842	0.880	0.861	0.723	0.923	0.858
	*M. musculus*	0.932	1	0.966	0.935	0.974	0.965
iDNA6mA-PseKNC	Cross-species	0.762	0.769	0.765	0.531	0.844	0.764
	Rice	0.569	0.721	0.641	0.394	0.896	0.543
	*M. musculus*	0.869	1	0.935	0.877	0.974	0.930

## Discussion

Unlike the prediction of m^6^A modifications in mRNA, the identification of 6 mA modifications in DNA is still at the beginning. In this study, we developed an improved tool, called csDMA, for predicting 6 mA modifications in different species. Three feature encoding strategies were used to generate the feature matrix and different algorithms were selected into the model. For performance evaluation, 5-fold cross-validation and independent test were used and the ExtraTrees classifier received the best performance on the training and independent test datasets. We also compared the performance of our tool with that of iDNA6mA-PseKNC. And the results showed that our model improved the recognition performance of DNA 6 mA modifications effectively.

The i6mA-Pred predictor is another of the two existing tools for DNA 6 mA prediction. However, the research paper is still in the corrected proof phase and their method cannot be reached until our work finished. Fortunately, we acknowledge from their online abstract that the method received an ACC of 0.831 by using a jackknife test. As jackknife test will generate a fixed ACC on the same dataset and their dataset was also downloaded as the *rice* dataset in this study. Thus, we also evaluated the performance of our model on the rice dataset by using a jackknife test and our model received an ACC of 0.859, which is also higher than that of i6mA-Pred.

Although our model received a high performance on the *M. musculus* dataset, the performance on the *rice* and cross-species datasets were relatively low. In the future, more feature encoding schemes, such as genomic and structural features, will be used to improve the performance of csDMA. And also we will extend csDMA to other species, such as *human* and *Arabidopsis thaliana*.

## Supplementary information

supplementary material

supplementary dataset
