# Supplementary material for: csDMA: an improved bioinformatics tool for identifying DNA 6 mA modifications via Chou’s 5-step rule
Source: Sci Rep. 2019 Sep 11;9:13109. doi: 10.1038/s41598-019-49430-4 (PMC6739324; doi:10.1038/s41598-019-49430-4)
Supplement: Supplementary file 1 — supplementary material [file 41598_2019_49430_MOESM1_ESM.pdf]

csDMA: an improved bioinformatics tool for identifying DNA 6mA modifications via Chou's 5-step rule

Ze Liu[1,2], Wei Dong[1,2*], Wei Jiang[1,2], Zili He[1,2]

[1]College of Water Resources and Architectural Engineering, Northwest A&F University, Yangling, 712100, Shaanxi, China. [2]Key Laboratory of Agricultural Soil and Water Engineering in Arid and Semiarid Areas, Ministry of Education, Northwest A & F University, Yangling, 712100, Shaanxi, China. Correspondence and requests for materials should be addressed to W.D. (email: dongw@nwafu.edu.cn)

****
MEME - Motif discovery tool
****
MEME version 5.0.5 (Release date: Mon Mar 18 20:12:19 2019 -0700)

For further information on how to interpret please access http://meme-suite.org/.
To get a copy of the MEME software please access http://meme-suite.org.

****

****
REFERENCE
****
If you use this program in your research, please cite:

Timothy L. Bailey and Charles Elkan,
"Fitting a mixture model by expectation maximization to discover
motifs in biopolymers", Proceedings of the Second International
Conference on Intelligent Systems for Molecular Biology, pp. 28-36,

AAAI Press, Menlo Park, California, 1994.
****

****
TRAINING SET
****
PRIMARY SEQUENCES= posdata.fa
CONTROL SEQUENCES= negdata.fa
ALPHABET= ACGT

****

****
COMMAND LINE SUMMARY
****
This information can also be useful in the event you wish to report a
problem with the MEME software.

command: meme posdata.fa -dna -oc . -nostatus -time 18000 -mod zoops -nmotifs 10 -minw 6 -maxw 50 -objfun de -neg negdata.fa -revcomp -markov_order 0

```
model:  mod=         zoops    nmotifs=        10    evt=           inf
objective function:           em=       Differential Enrichment mHG
                              starts=   log likelihood ratio (LLR)
strands: + -
width:  minw=            6    maxw=           41
nsites: minsites=        2    maxsites=     2814    wnsites=       0.8
theta:  spmap=         uni    spfuzz=        0.5
em:     prior=   dirichlet    b=            0.01    maxiter=        50
        distance=    1e-05
data:   n=          115374    N=            2814
sample: seed=            0    hsfrac=        0.5
        searchsize= 100000    norand=         no    csites=         -1
```
Letter frequencies in dataset:
A 0.29 C 0.21 G 0.21 T 0.29
Background letter frequencies (from file dataset with add-one prior applied):
A 0.29 C 0.21 G 0.21 T 0.29

Background model order: 0
****

****
MOTIF NNNNNNNHHNHHNHWNTNTNWNNNWNYNNNNNNNNNNNNNN MEME-1 width = 41 sites = 1537 llr = 2837 p-value = 3.3e-018 E-value = 3.3e-018
****

Motif NNNNNNNHHNHHNHWNTNTNWNNNWNYNNNNNNNNNNNNNN MEME-1 Description
----
Simplified A 22222222222222223223242223223222232222322232332
pos.-specific C 22322222222222223:3:2222232222222222222
probability G 22222222222222222:2:332222222222222222222
matrix T 43433334444444434373633444434443333333333

bits 2.3
2.0
1.8
1.6
Relative 1.4
Entropy 1.1
(2.7 bits) 0.9 * *
0.7 * *
0.5 * *
0.2 * *
0.0 ---

Multilevel TTTTTTTTTTTTTTTTTCTTTTTTTTTTTTTTTTTTTTTTT
consensus
CACCAAACAACAACAAATACAGGCACCAACAAAAAAAAAAC
sequence ACAACCCACCACGA G A G CC G GC CCCCCCGCCCA
G GG A G GG G GG

Motif NNNNNNNHHNHHNHWNTNTNWNNNWNYNNNNNNNNNNNNNN MEME-1 position-specific scoring matrix
----

log-odds matrix: alength= 4 w= 41 n= 2814 bayes= 1.71048 E= 3.3e-018

-37	10	-9	29
-29	10	2	17
-51	26	-15	29
-31	17	-11	21
-27	11	-20	27
-33	10	-5	24
-30	6	-7	25
-30	17	-31	31
-27	4	-32	37
-28	14	-21	27
-37	17	-29	34
-29	1	-27	38
-30	-15	-3	35
-42	21	-24	32
-44	-7	-24	49
-13	-12	-2	20
-48	-16	-22	55
-27	48	-17	-5
18	-1723	-1723	121
-56	34	24	0
60	-1723	-1723	95
-57	0	41	13
-54	9	26	17
-56	9	-11	41
-5	-23	-33	37
-49	11	2	29
-72	45	-47	41
-10	-10	-6	20
-34	-5	-13	38
-58	23	-9	32
-32	0	-11	33
-24	0	-6	24
-14	18	-18	12
-28	0	-5	27
-27	12	-3	17
-25	8	-12	24
-13	-8	-2	19
-24	15	1	10
-20	14	-14	17
-11	1	0	10
-36	12	2	20

Motif NNNNNNNHHNHHNHWNTNTNWNNNWNYNNNNNNNNNNNNNN MEME-1 position-specific probability matrix
----
letter-probability matrix: alength= 4 w= 41 nsites= 1537 E= 3.3e-018

0.223813	0.224463	0.197788	0.353936
0.236825	0.224463	0.212752	0.325960
0.204294	0.251789	0.188679	0.355237
0.234223	0.236825	0.194535	0.334418
0.240729	0.227066	0.182824	0.349382
0.230319	0.225114	0.202993	0.341574
0.235524	0.218608	0.200390	0.345478
0.234873	0.236825	0.169811	0.358491
0.240078	0.216005	0.167859	0.376057
0.238126	0.230969	0.180872	0.350033
0.225114	0.236174	0.171763	0.366949
0.237476	0.211451	0.173715	0.377358
0.235524	0.188679	0.205595	0.370202
0.217306	0.243331	0.177619	0.361744
0.214053	0.199740	0.178269	0.407938
0.265452	0.193234	0.207547	0.333767
0.207547	0.188029	0.180221	0.424203
0.240078	0.293429	0.186727	0.279766
0.327912	0.000000	0.000000	0.672088
0.196487	0.266103	0.247235	0.290176
0.439818	0.000000	0.000000	0.560182
0.195185	0.209499	0.278465	0.316851
0.199089	0.223162	0.251789	0.325960
0.197137	0.223813	0.194535	0.384515
0.279766	0.178920	0.167209	0.374105
0.206897	0.226415	0.212752	0.353936
0.175667	0.287573	0.151594	0.385166
0.270007	0.195185	0.201041	0.333767
0.229668	0.202342	0.191282	0.376708
0.194535	0.245934	0.197137	0.362394
0.231620	0.209499	0.194535	0.364346
0.245283	0.210150	0.201041	0.343526
0.262850	0.237476	0.184776	0.314899
0.238126	0.209499	0.202993	0.349382
0.240078	0.227716	0.204945	0.327261
0.243331	0.221210	0.192583	0.342876
0.264802	0.198439	0.206897	0.329863
0.245934	0.232921	0.210800	0.310345
0.251789	0.230969	0.189980	0.327261

0.268705   0.210800   0.209499   0.310995
0.226415   0.227716   0.212752   0.333116

Motif NNNNNNNHHNHHNHWNTNTNWNNNWNYNNNNNNNNNNNNNN MEME-1 regular expression
----
[TCA][TACG][TCA][TCA][TAC][TACG][TACG][TCA][TAC][TAC][TCA][TAC][TAG][TCA][TA][TAG][TA][CTA][TA][TCG][TA][TGC][TGC][TC][TA][TCGA][TC][TAG][TAC][TC][TAC][TACG][TAC][TACG][TACG][TAC][TAG][TACG][TAC][TACG][TCAG]

Time 694.83 secs.

****

****
MOTIF TYTTKTWWTTTWAARAAA MEME-2   width = 18   sites = 34   llr = 349   p-value = 8.8e-001   E-value = 1.8e+000
****

Motif TYTTKTWWTTTWAARAAA MEME-2 Description

```
Simplified        A   ::2:2:63::15675599
pos.-specific     C   :5:::1:::1211::1::
probability       G   1::142:2222:1:52::
matrix            T   95893645886413:21:

         bits     2.3
                  2.0
                  1.8
                  1.6
Relative          1.4 *   *           **
Entropy           1.1 *   *     *     * **
(14.8 bits)       0.9 ****   * **     * **
```

0.7 **** ** **   ** **
0.5 *************** **
0.2 ******************
0.0 -----------------

Multilevel TTTTGTATTTTAAAGAAA
consensus CA TGTAG T TAG
sequence A G

Motif TYTTKTWWTTTWAARAAA MEME-2 position-specific scoring matrix
----
log-odds matrix: alength= 4 w= 18 n= 67536 bayes= 11.4821 E= 1.8e+000

-1173	-1173	-125	165
-1173	116	-1173	87
-49	-283	-1173	140
-1173	-1173	-83	160
-49	-283	107	16
-1173	-51	16	109
109	-1173	-1173	40
-13	-1173	16	79
-1173	-1173	-3	145
-1173	-183	-25	140
-230	-25	-25	102
87	-125	-283	28
116	-51	-125	-130
122	-1173	-283	2
70	-1173	133	-1173
87	-125	-3	-72
165	-1173	-1173	-172
170	-1173	-283	-330

Motif TYTTKTWWTTTWAARAAA MEME-2 position-specific probability matrix
----
letter-probability matrix: alength= 4 w= 18 nsites= 34 E= 1.8e+000

0.000000	0.000000	0.088235	0.911765
0.000000	0.470588	0.000000	0.529412
0.205882	0.029412	0.000000	0.764706
0.000000	0.000000	0.117647	0.882353

0.205882	0.029412	0.441176	0.323529
0.000000	0.147059	0.235294	0.617647
0.617647	0.000000	0.000000	0.382353
0.264706	0.000000	0.235294	0.500000
0.000000	0.000000	0.205882	0.794118
0.000000	0.058824	0.176471	0.764706
0.058824	0.176471	0.176471	0.588235
0.529412	0.088235	0.029412	0.352941
0.647059	0.147059	0.088235	0.117647
0.676471	0.000000	0.029412	0.294118
0.470588	0.000000	0.529412	0.000000
0.529412	0.088235	0.205882	0.176471
0.911765	0.000000	0.000000	0.088235
0.941176	0.000000	0.029412	0.029412

Motif TYTTKTWWTTTWAARAAA MEME-2 regular expression

T[TC][TA]T[GTA][TG][AT][TAG][TG]TT[AT]A[AT][GA][AG]AA

Time 1154.91 secs.

****

****

MOTIF ACCGATCSA MEME-3 width = 9 sites = 22 llr = 208 p-value = 2.8e-002 E-value = 2.9e-002

****

Motif ACCGATCSA MEME-3 Description

Simplified A a:::71:2a
pos.-specific C :8a3::a4:
probability G :2:72::4:
matrix T :::::9:::

```
           bits    2.3    *    *
                    2.0    *    *
                    1.8    *    * *
                    1.6 ***    * *
Relative           1.4 **** ** *
Entropy             1.1 **** ** *
(13.6 bits)       0.9 ******* *
                    0.7 *********
                    0.5 *********
                    0.2 *********
                    0.0 ---------

Multilevel           ACCGATCCA
consensus              CG   G
sequence
```

Motif ACCGATCSA MEME-3 position-specific scoring matrix

----

log-odds matrix: alength= 4 w= 9 n= 92862 bayes= 12.9666 E= 2.9e-002

172	-220	-1110	-1110
-1110	196	-21	-1110
-1110	225	-1110	-1110
-1110	60	170	-1110
133	-220	11	-1110
-167	-1110	-1110	165
-1110	225	-1110	-1110
-67	96	96	-1110
178	-1110	-1110	-1110

Motif ACCGATCSA MEME-3 position-specific probability matrix

----

letter-probability matrix: alength= 4 w= 9 nsites= 22 E= 2.9e-002

0.954545	0.045455	0.000000	0.000000
0.000000	0.818182	0.181818	0.000000
0.000000	1.000000	0.000000	0.000000
0.000000	0.318182	0.681818	0.000000
0.727273	0.045455	0.227273	0.000000
0.090909	0.000000	0.000000	0.909091

0.000000 1.000000 0.000000 0.000000
0.181818 0.409091 0.409091 0.000000
1.000000 0.000000 0.000000 0.000000

Motif ACCGATCSA MEME-3 regular expression

ACC[GC][AG]TC[CG]A

Time 1590.17 secs.

****

****
MOTIF AAAAAAAAAVMA MEME-4 width = 12 sites = 31 llr = 311 p-value = 9.8e-001 E-value = 3.1e+000
****

Motif AAAAAAAAAVMA MEME-4 Description

```
Simplified        A  a87a867aa35a
pos.-specific     C  :2:::23::44:
probability       G  ::2::2:::3::
matrix            T  ::1:2::::1:

         bits    2.3
                 2.0
                 1.8 *       **   *
                 1.6 *   *   **   *
Relative         1.4 *   *   **   *
Entropy          1.1 ** ** ***   *
(14.5 bits)      0.9 ** ** ***   *
                 0.7 ********* **
                 0.5 ************
                 0.2 ************
```

0.0 -----------

Multilevel AAAAAAAAACAA
consensus GC AC
sequence G

Motif AAAAAAAAAVMA MEME-4 position-specific scoring matrix
----
log-odds matrix: alength= 4 w= 12 n= 84420 bayes= 12.4582 E= 3.1e+000

179	-1160	-1160	-1160
147	-12	-1160	-1160
122	-1160	-12	-117
174	-270	-1160	-1160
147	-1160	-1160	-58
108	-38	11	-1160
122	62	-1160	-1160
179	-1160	-1160	-1160
179	-1160	-1160	-1160
15	100	30	-1160
92	76	-1160	-158
179	-1160	-1160	-1160

Motif AAAAAAAAAVMA MEME-4 position-specific probability matrix
----
letter-probability matrix: alength= 4 w= 12 nsites= 31 E= 3.1e+000

1.000000	0.000000	0.000000	0.000000
0.806452	0.193548	0.000000	0.000000
0.677419	0.000000	0.193548	0.129032
0.967742	0.032258	0.000000	0.000000
0.806452	0.000000	0.000000	0.193548
0.612903	0.161290	0.225806	0.000000
0.677419	0.322581	0.000000	0.000000
1.000000	0.000000	0.000000	0.000000
1.000000	0.000000	0.000000	0.000000
0.322581	0.419355	0.258065	0.000000
0.548387	0.354839	0.000000	0.096774
1.000000	0.000000	0.000000	0.000000

```
--------------------------------------------------------------------------------
	Motif AAAAAAAAAVMA MEME-4 regular expression
--------------------------------------------------------------------------------
AAAAA[AG][AC]AA[CAG][AC]A
--------------------------------------------------------------------------------




Time 2032.58 secs.

********************************************************************************


********************************************************************************
MOTIF SKSRVVMGDSVGVGSVAGGYRSCRGSNVYGBGBGG MEME-5	width =  35  sites =   8  llr = 153  p-value = 9.8e-001  E-value = 3.0e+000
********************************************************************************
--------------------------------------------------------------------------------
	Motif SKSRVVMGDSVGVGSVAGGYRSCRGSNVYGBGBGG MEME-5 Description
--------------------------------------------------------------------------------
Simplified        A  11:4444:3:3131:3a1:161:51:3311::11:
pos.-specific     C  51413341:63:3363::13:4a::5335:41313
probability       G  4555331954464645:99145:484341946466
matrix            T  :31:111:3:131::::::5:::111313:33311

          bits    2.3                  *
                  2.0                   *
                  1.8        *      * *  *
                  1.6        *      ***  *     *
Relative          1.4       * *   * ***  *     *
Entropy           1.1       * *   * ***  * *   *
(27.5 bits)       0.9      * *  ** *** * * *   * *  *
                  0.7 * **  * * * ****** *** **   *** **
                  0.5 * **  *** * ****** ******   *** **
                  0.2 ************************** ********
                  0.0 -----------------------------------

Multilevel           CGGGAAAGGCGGGGCGAGGTAGCAGCAGCGCGGGG
consensus             GTCACCC AGATACGA   CGC G GCAT GTC C
```

sequence GG T C C C GC T T
T

Motif SKSRVVMGDSVGVGSVAGGYRSCRGSNVYGBGBGG MEME-5 position-specific scoring matrix

----

log-odds matrix: alength= 4 w= 35 n= 19698 bayes= 9.33753 E= 3.0e+000

-121	125	84	-965
-121	-75	125	-21
-965	84	125	-121
37	-75	125	-965
37	25	25	-121
37	25	25	-121
37	84	-75	-121
-965	-75	206	-965
-21	-965	125	-21
-965	157	84	-965
-21	25	84	-121
-121	-965	157	-21
-21	25	84	-121
-121	25	157	-965
-965	157	84	-965
-21	25	125	-965
178	-965	-965	-965
-121	-965	206	-965
-965	-75	206	-965
-121	25	-75	78
111	-965	84	-965
-121	84	125	-965
-965	225	-965	-965
78	-965	84	-121
-121	-965	184	-121
-965	125	84	-121
-21	25	25	-21
-21	25	84	-121
-121	125	-75	-21
-121	-965	206	-965
-965	84	84	-21
-965	-75	157	-21
-121	25	84	-21
-121	-75	157	-121
-965	25	157	-121

Motif SKSRVVMGDSVGVGSVAGGYRSCRGSNVYGBGBGG MEME-5 position-specific probability matrix

----

letter-probability matrix: alength= 4 w= 35 nsites= 8 E= 3.0e+000

0.125000	0.500000	0.375000	0.000000
0.125000	0.125000	0.500000	0.250000
0.000000	0.375000	0.500000	0.125000
0.375000	0.125000	0.500000	0.000000
0.375000	0.250000	0.250000	0.125000
0.375000	0.250000	0.250000	0.125000
0.375000	0.375000	0.125000	0.125000
0.000000	0.125000	0.875000	0.000000
0.250000	0.000000	0.500000	0.250000
0.000000	0.625000	0.375000	0.000000
0.250000	0.250000	0.375000	0.125000
0.125000	0.000000	0.625000	0.250000
0.250000	0.250000	0.375000	0.125000
0.125000	0.250000	0.625000	0.000000
0.000000	0.625000	0.375000	0.000000
0.250000	0.250000	0.500000	0.000000
1.000000	0.000000	0.000000	0.000000
0.125000	0.000000	0.875000	0.000000
0.000000	0.125000	0.875000	0.000000
0.125000	0.250000	0.125000	0.500000
0.625000	0.000000	0.375000	0.000000
0.125000	0.375000	0.500000	0.000000
0.000000	1.000000	0.000000	0.000000
0.500000	0.000000	0.375000	0.125000
0.125000	0.000000	0.750000	0.125000
0.000000	0.500000	0.375000	0.125000
0.250000	0.250000	0.250000	0.250000
0.250000	0.250000	0.375000	0.125000
0.125000	0.500000	0.125000	0.250000
0.125000	0.000000	0.875000	0.000000
0.000000	0.375000	0.375000	0.250000
0.000000	0.125000	0.625000	0.250000
0.125000	0.250000	0.375000	0.250000
0.125000	0.125000	0.625000	0.125000
0.000000	0.250000	0.625000	0.125000

Motif SKSRVVMGDSVGVGSVAGGYRSCRGSNVYGBGBGG MEME-5 regular expression
----
[CG][GT][GC][GA][ACG][ACG][AC]G[GAT][CG][GAC][GT][GAC][GC][CG][GAC]AGG[TC][AG][GC]C[AG]G[CG][ACGT][GAC][CT]G[CGT][GT][GCT]G[GC]

Time 2462.29 secs.

****

****
MOTIF TAAAWTAADKAVTTACAA MEME-6 width = 18 sites = 10 llr = 125 p-value = 1.0e+000 E-value = 4.4e+000
****

Motif TAAAWTAADKAVTTACAA MEME-6 Description

```
Simplified        A  :96851963:732:7:a8
pos.-specific     C  ::2222:21:33::3a::
probability       G  :::::1:234:412::::
matrix            T  a12:361:36::78:::2

         bits    2.3                   *
                 2.0                    *
                 1.8 *                 **
                 1.6 *                 **
Relative         1.4 **      *        **
Entropy          1.1 ** *    *    *   *****
(18.1 bits)      0.9 ** *    *   **   *****
                 0.7 ** *    ** ** ******
                 0.5 ******** *********
                 0.2 ******** *********
                 0.0 ------------------

Multilevel           TAAAATAAATAGTTACAA
```

consensus CCTC CGGCAAGC T
sequence T C GT C

Motif TAAAWTAADKAVTTACAA MEME-6 position-specific scoring matrix

---

log-odds matrix: alength= 4 w= 18 n= 67536 bayes= 12.7212 E= 4.4e+000

-997	-997	-997	178
163	-997	-997	-153
105	-7	-997	-54
146	-7	-997	-997
78	-7	-997	5
-153	-7	-107	105
163	-997	-997	-153
105	-7	-7	-997
5	-107	51	5
-997	-997	93	105
127	51	-997	-997
5	51	93	-997
-54	-997	-107	127
-997	-997	-7	146
127	51	-997	-997
-997	225	-997	-997
178	-997	-997	-997
146	-997	-997	-54

Motif TAAAWTAADKAVTTACAA MEME-6 position-specific probability matrix

---

letter-probability matrix: alength= 4 w= 18 nsites= 10 E= 4.4e+000

0.000000	0.000000	0.000000	1.000000
0.900000	0.000000	0.000000	0.100000
0.600000	0.200000	0.000000	0.200000
0.800000	0.200000	0.000000	0.000000
0.500000	0.200000	0.000000	0.300000
0.100000	0.200000	0.100000	0.600000
0.900000	0.000000	0.000000	0.100000
0.600000	0.200000	0.200000	0.000000
0.300000	0.100000	0.300000	0.300000
0.000000	0.000000	0.400000	0.600000
0.700000	0.300000	0.000000	0.000000

```
 0.300000   0.300000   0.400000   0.000000
 0.200000   0.000000   0.100000   0.700000
 0.000000   0.000000   0.200000   0.800000
 0.700000   0.300000   0.000000   0.000000
 0.000000   1.000000   0.000000   0.000000
 1.000000   0.000000   0.000000   0.000000
 0.800000   0.000000   0.000000   0.200000
--------------------------------------------------------------------------------

--------------------------------------------------------------------------------
	Motif TAAAWTAADKAVTTACAA MEME-6 regular expression
--------------------------------------------------------------------------------
TA[ACT][AC][ATC][TC]A[ACG][AGT][TG][AC][GAC][TA][TG][AC]CA[AT]
--------------------------------------------------------------------------------




Time 2877.17 secs.

********************************************************************************


********************************************************************************
MOTIF AVCTGRMAMTSATGG MEME-7 width =   15   sites =   16   llr = 199   p-value = 1.0e+000   E-value = 4.5e+000
********************************************************************************
--------------------------------------------------------------------------------
	Motif AVCTGRMAMTSATGG MEME-7 Description
--------------------------------------------------------------------------------
Simplified        A  93:::4584316::1
pos.-specific     C  :4a2:2526141:::
probability       G  :3::a4::::432a9
matrix            T  1::8:::1:71:8::

         bits    2.3     * *           *
                 2.0     * *           *
                 1.8     * *           *
                 1.6     * *           **
Relative         1.4 * * *          **
Entropy          1.1 * *** * *     ***
```

```
(17.9 bits)          0.9 * *** ***      ***
                        0.7 * *** **** ****
                        0.5 ***************
                        0.2 ***************
                        0.0 ---------------

Multilevel                 ACCTGAAACTGATGG
consensus                    A     GC AACG
sequence                     G
```

Motif AVCTGRMAMTSATGG MEME-7 position-specific scoring matrix

----

log-odds matrix: alength= 4 w= 15 n= 75978 bayes= 12.213 E= 4.5e+000

```
   159  -1064  -1064   -121
    11    106     25  -1064
 -1064    225  -1064  -1064
 -1064    -16  -1064    149
 -1064  -1064    225  -1064
    59    -16     84  -1064
    78    125  -1064  -1064
   137    -16  -1064   -221
    37    157  -1064  -1064
   -21   -175  -1064    124
  -121     84    106   -221
   111    -75     25  -1064
 -1064  -1064    -16    149
 -1064  -1064    225  -1064
  -121  -1064    206  -1064
```

Motif AVCTGRMAMTSATGG MEME-7 position-specific probability matrix

----

letter-probability matrix: alength= 4 w= 15 nsites= 16 E= 4.5e+000

```
 0.875000  0.000000  0.000000  0.125000
 0.312500  0.437500  0.250000  0.000000
 0.000000  1.000000  0.000000  0.000000
 0.000000  0.187500  0.000000  0.812500
 0.000000  0.000000  1.000000  0.000000
 0.437500  0.187500  0.375000  0.000000
 0.500000  0.500000  0.000000  0.000000
```

0.750000	0.187500	0.000000	0.062500
0.375000	0.625000	0.000000	0.000000
0.250000	0.062500	0.000000	0.687500
0.125000	0.375000	0.437500	0.062500
0.625000	0.125000	0.250000	0.000000
0.000000	0.000000	0.187500	0.812500
0.000000	0.000000	1.000000	0.000000
0.125000	0.000000	0.875000	0.000000

Motif AVCTGRMAMTSATGG MEME-7 regular expression

A[CAG]CTG[AG][AC]A[CA][TA][GC][AG]TGG

Time 3302.72 secs.

****

****

MOTIF CTCCCAADGAAARRRTCWYAAR MEME-8 width = 22 sites = 11 llr = 151 p-value = 1.0e+000 E-value = 6.6e+000

****

Motif CTCCCAADGAAARRRTCWYAAR MEME-8 Description

```
Simplified        A  ::2118642a985541:3:874
pos.-specific     C  7276823:2:::1::3726:::
probability       G  11::::45:11445:1::236
matrix            T  27131:132::11126254:::

          bits 2.3
               2.0
               1.8        *
               1.6        *
Relative       1.4    *   **
```

```
Entropy          1.1 * * **      **         * ****
(19.8 bits)      0.9 ******      ***        * ****
                 0.7 *******    *** * ** ****
                 0.5 ******** *** *********
                 0.2 *********************
                 0.0 ---------------------

Multilevel           CTCCCAAAGAAAAAGTCTCAAG
consensus                T    CG       GGAC AT GA
sequence                      T
```

Motif CTCCCAADGAAARRRTCWYAAR MEME-8 position-specific scoring matrix

----

log-odds matrix: alength= 4 w= 22 n= 56280 bayes= 12.5474 E= 6.6e+000

-1010	179	-121	-67
-1010	-21	-121	133
-67	179	-1010	-167
-167	160	-1010	-9
-167	196	-1010	-167
150	-21	-1010	-1010
113	38	-1010	-167
33	-1010	79	-9
-67	-21	111	-67
178	-1010	-1010	-1010
165	-1010	-121	-1010
150	-1010	-121	-167
65	-121	79	-167
91	-1010	79	-167
33	-1010	111	-67
-167	38	-1010	113
-1010	179	-121	-67
-9	-21	-1010	91
-1010	160	-1010	33
150	-1010	-21	-1010
133	-1010	38	-1010
33	-1010	160	-1010

Motif CTCCCAADGAAARRRTCWYAAR MEME-8 position-specific probability

matrix

----

letter-probability matrix: alength= 4 w= 22 nsites= 11 E= 6.6e+000

0.000000	0.727273	0.090909	0.181818
0.000000	0.181818	0.090909	0.727273
0.181818	0.727273	0.000000	0.090909
0.090909	0.636364	0.000000	0.272727
0.090909	0.818182	0.000000	0.090909
0.818182	0.181818	0.000000	0.000000
0.636364	0.272727	0.000000	0.090909
0.363636	0.000000	0.363636	0.272727
0.181818	0.181818	0.454545	0.181818
1.000000	0.000000	0.000000	0.000000
0.909091	0.000000	0.090909	0.000000
0.818182	0.000000	0.090909	0.090909
0.454545	0.090909	0.363636	0.090909
0.545455	0.000000	0.363636	0.090909
0.363636	0.000000	0.454545	0.181818
0.090909	0.272727	0.000000	0.636364
0.000000	0.727273	0.090909	0.181818
0.272727	0.181818	0.000000	0.545455
0.000000	0.636364	0.000000	0.363636
0.818182	0.000000	0.181818	0.000000
0.727273	0.000000	0.272727	0.000000
0.363636	0.000000	0.636364	0.000000

Motif CTCCCAADGAAARRRTCWYAAR MEME-8 regular expression

CTC[CT]CA[AC][AGT]GAAA[AG][AG][GA][TC]C[TA][CT]A[AG][GA]

Time 3724.89 secs.

****

****

MOTIF TGTCHCTDTRCC MEME-9 width = 12 sites = 12 llr = 133 p-value = 1.0e+000 E-value = 3.8e+000
****

```
--------------------------------------------------------------------------------
	Motif TGTCHCTDTRCC MEME-9 Description
--------------------------------------------------------------------------------
Simplified        A  ::::3::3:3::
pos.-specific     C  1:395a2:::99
probability       G  :9:::::525::
matrix            T  91813:838211

         bits    2.3     *
                 2.0      *
                 1.8   * * *    **
                 1.6   * * *    **
Relative         1.4 ** * *    **
Entropy          1.1 **** ** * **
(16.0 bits)      0.9 **** ** * **
                 0.7 **** ** * **
                 0.5 ************
                 0.2 ************
                 0.0 ------------

Multilevel           TGTCCCTGTGCC
consensus              C A  A A
sequence                T  T

--------------------------------------------------------------------------------


--------------------------------------------------------------------------------
	Motif TGTCHCTDTRCC MEME-9 position-specific scoring matrix
--------------------------------------------------------------------------------
log-odds matrix: alength= 4 w= 12 n= 84420 bayes= 12.7801 E= 3.8e+000
 -1023   -133  -1023    166
 -1023  -1023    213   -180
 -1023     25  -1023    137
 -1023    213  -1023   -180
   -21    125  -1023    -21
 -1023    225  -1023  -1023
 -1023    -33  -1023    152
   -21  -1023    125    -21
 -1023  -1023    -33    152
    20  -1023    125    -80
```

-1023 213 -1023 -180
-1023 213 -1023 -180

Motif TGTCHCTDTRCC MEME-9 position-specific probability matrix
----
letter-probability matrix: alength= 4 w= 12 nsites= 12 E= 3.8e+000

0.000000	0.083333	0.000000	0.916667
0.000000	0.000000	0.916667	0.083333
0.000000	0.250000	0.000000	0.750000
0.000000	0.916667	0.000000	0.083333
0.250000	0.500000	0.000000	0.250000
0.000000	1.000000	0.000000	0.000000
0.000000	0.166667	0.000000	0.833333
0.250000	0.000000	0.500000	0.250000
0.000000	0.000000	0.166667	0.833333
0.333333	0.000000	0.500000	0.166667
0.000000	0.916667	0.000000	0.083333
0.000000	0.916667	0.000000	0.083333

Motif TGTCHCTDTRCC MEME-9 regular expression

TG[TC]C[CAT]CT[GAT]T[GA]CC

Time 4115.84 secs.

****

****
MOTIF MAMAMCWACYTARTAVTARMWGTGAYW MEME-10 width = 27 sites = 13 llr = 187 p-value = 1.0e+000 E-value = 7.0e+000
****

Motif MAMAMCWACYTARTAVTARMWGTGAYW MEME-10 Description

```
Simplified        A  5a674:6811183284276332226:5
pos.-specific     C  4:4248:285:222:31::521:124:
probability       G  :::212::1:2:4:22224::827222
matrix            T  2:::2:4:147:261151:25:6::53

          bits    2.3
                  2.0
                  1.8    *
                  1.6    *     *
Relative          1.4   *     *
Entropy           1.1    *     * **   *           *
(20.8 bits)       0.9  **   ****   *  *    *   * *
                  0.7    *** *******   *    *** * ***
                  0.5 **** ******* **    **********
                  0.2 ***************************
                  0.0 ---------------------------

Multilevel            AAAAACAACCTAGTAATAACTGTGATA
consensus              C C C T   TGCAA CGGGAA AACCT
sequence                                 G
```

Motif MAMAMCWACYTARTAVTARMWGTGAYW MEME-10 position-specific scoring matrix

----

log-odds matrix: alength= 4 w= 27 n= 42210 bayes= 12.0722 E= 7.0e+000

67	87	-1035	-91
178	-1035	-1035	-1035
108	87	-1035	-1035
125	-45	-45	-1035
41	87	-145	-91
-1035	201	-45	-1035
108	-1035	-1035	41
154	-45	-1035	-1035
-191	187	-145	-191
-191	136	-1035	41
-191	-1035	14	125
141	14	-1035	-1035
8	-45	87	-91
-33	-45	-1035	108

141	-1035	-45	-191
41	55	14	-191
-91	-145	14	89
125	-1035	14	-191
108	-1035	87	-1035
8	136	-1035	-91
8	-45	-1035	89
-91	-145	187	-1035
-33	-1035	-45	108
-33	-145	172	-1035
108	14	-45	-1035
-1035	87	-45	67
89	-1035	-45	8

Motif MAMAMCWACYTARTAVTARMWGTGAYW MEME-10 position-specific probability matrix

----

letter-probability matrix: alength= 4 w= 27 nsites= 13 E= 7.0e+000

0.461538	0.384615	0.000000	0.153846
1.000000	0.000000	0.000000	0.000000
0.615385	0.384615	0.000000	0.000000
0.692308	0.153846	0.153846	0.000000
0.384615	0.384615	0.076923	0.153846
0.000000	0.846154	0.153846	0.000000
0.615385	0.000000	0.000000	0.384615
0.846154	0.153846	0.000000	0.000000
0.076923	0.769231	0.076923	0.076923
0.076923	0.538462	0.000000	0.384615
0.076923	0.000000	0.230769	0.692308
0.769231	0.230769	0.000000	0.000000
0.307692	0.153846	0.384615	0.153846
0.230769	0.153846	0.000000	0.615385
0.769231	0.000000	0.153846	0.076923
0.384615	0.307692	0.230769	0.076923
0.153846	0.076923	0.230769	0.538462
0.692308	0.000000	0.230769	0.076923
0.615385	0.000000	0.384615	0.000000
0.307692	0.538462	0.000000	0.153846
0.307692	0.153846	0.000000	0.538462
0.153846	0.076923	0.769231	0.000000
0.230769	0.000000	0.153846	0.615385
0.230769	0.076923	0.692308	0.000000

0.615385 0.230769 0.153846 0.000000
0.000000 0.384615 0.153846 0.461538
0.538462 0.000000 0.153846 0.307692

Motif MAMAMCWACYTARTAVTARMWGTGAYW MEME-10 regular expression

[AC]A[AC]A[AC]C[AT]AC[CT][TG][AC][GA][TA]A[ACG][TG][AG][AG][CA][TA]G[TA][GA][AC][TC][AT]

Time 4498.80 secs.

****

****
Stopped because requested number of motifs (10) found.
****

CPU: node058

****
